# Coupling Between Production of Ribosomal RNA and Maturation: Just at the Beginning

**DOI:** 10.3389/fmolb.2021.778778

**Published:** 2021-10-26

**Authors:** Chaima Azouzi, Mariam Jaafar, Christophe Dez, Raghida Abou Merhi, Annick Lesne, Anthony K. Henras, Olivier Gadal

**Affiliations:** ^1^ Laboratoire de Biologie Moléculaire, Cellulaire et du Développement (MCD), Centre de Biologie Intégrative (CBI), CNRS, UPS, Université de Toulouse, Toulouse, France; ^2^ Genomic Stability and Biotherapy (GSBT) Laboratory, Faculty of Sciences, Rafik Hariri Campus, Lebanese University, Beirut, Lebanon; ^3^ CNRS, Laboratoire de Physique Théorique de la Matière Condensée, LPTMC, Sorbonne Université, Paris, France; ^4^ Institut de Génétique Moléculaire de Montpellier, IGMM, CNRS, Université Montpellier, Montpellier, France

**Keywords:** RNA polymerase I (Pol I), ribosomal RNA (rRNA) processing, transcription, termination of transcription, ribosomal RNA (rRNA) genes, RNA folding, premature termination of transcription

## Abstract

Ribosomal RNA (rRNA) production represents the most active transcription in the cell. Synthesis of the large rRNA precursors (35S/47S in yeast/human) is achieved by up to hundreds of RNA polymerase I (Pol I) enzymes simultaneously transcribing a single rRNA gene. In this review, we present recent advances in understanding the coupling between rRNA production and nascent rRNA folding. Mapping of the distribution of Pol I along ribosomal DNA at nucleotide resolution, using either native elongating transcript sequencing (NET-Seq) or crosslinking and analysis of cDNAs (CRAC), revealed frequent Pol I pausing, and CRAC results revealed a direct coupling between pausing and nascent RNA folding. High density of Pol I per gene imposes topological constraints that establish a defined pattern of polymerase distribution along the gene, with a persistent spacing between transcribing enzymes. RNA folding during transcription directly acts as an anti-pausing mechanism, implying that proper folding of the nascent rRNA favors elongation *in vivo*. Defects in co-transcriptional folding of rRNA are likely to induce Pol I pausing. We propose that premature termination of transcription, at defined positions, can control rRNA production *in vivo*.

## Synthesis of the 35S Primary Transcript by Pol I

**FIGURE 1:**
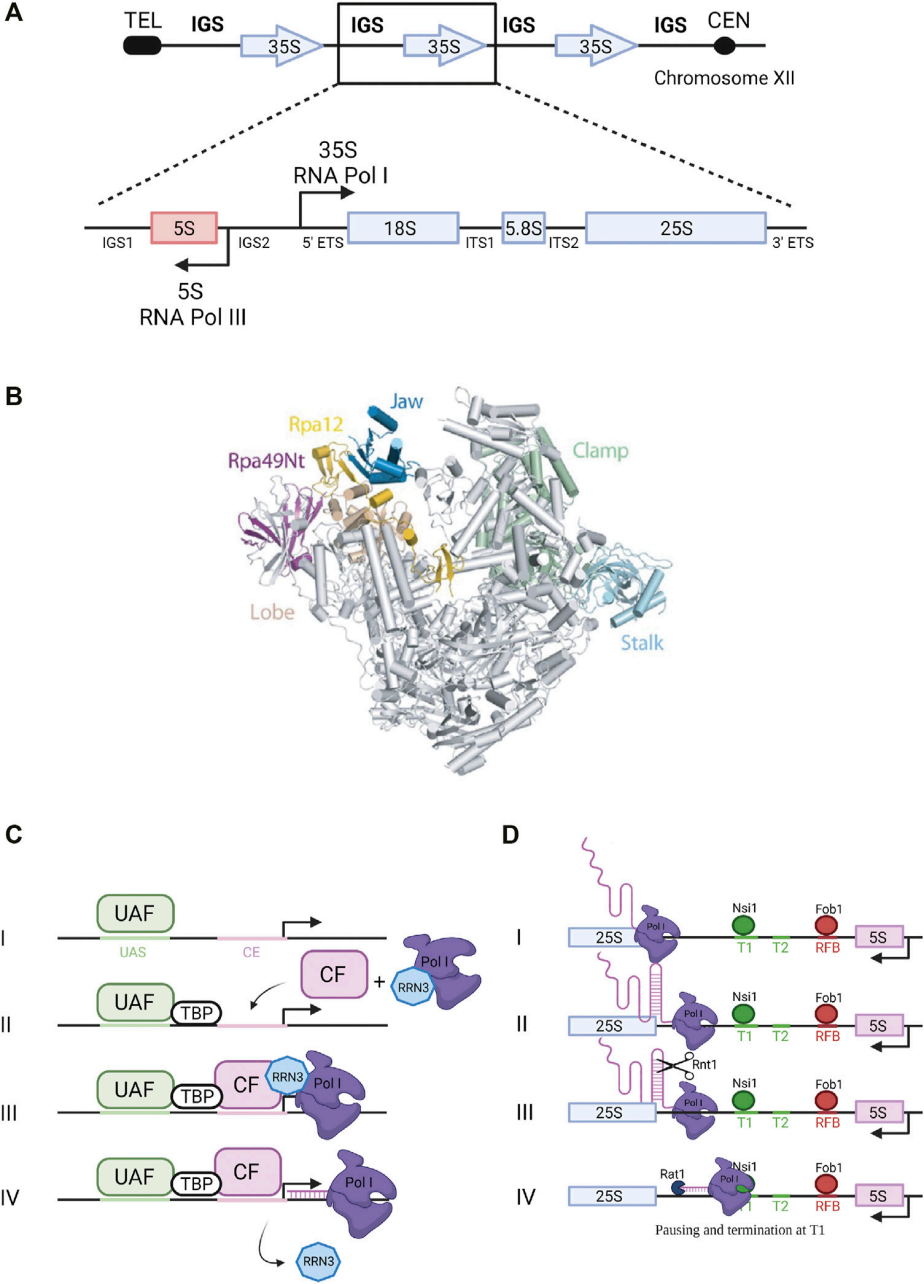
Ribosomal DNA transcription by RNA Pol I. **(A)** Ribosomal DNA. The rDNA repeats (150–200 copies) are located on chromosome XII. A single repeated unit is transcribed by RNA polymerase I (Pol I) to synthesize the 35S primary pre-rRNA transcript, which is then processed to produce the mature 18S, 5.8S and 25S rRNAs (arrow pointing to the right). RNA Polymerase III synthesizes the 5S rRNA (arrow pointing to the left). IGS, intergenic sequence; ETS, external transcribed spacer; ITS, internal transcribed spacer. **(B)** RNA Polymerase I. Pol I 3D structure (Darrière et al., 2019). View of the initially transcribing complex model and its four different subunits - PDB 5W66 (Han et al., 2017). Catalytic amino acids are located in the center of the central cleft. The two main modules are mobile and allow cleft opening and closure, depending of the transcription step. **(C)** Transcription initiation. Composition of Pol I pre-initiation complex (*see* text for details). UAF, Upstream Activating Factor; TBP, TATA-binding protein; CF, Core Factor. **(D)** Transcription termination. Pol I termination mechanisms (*see* text for details).

Yeast haploid cells contain between 150 and 200 copies of tandemly repeated rRNA genes while the diploid human genome contains around 400 copies. Although present at a high copy number in the genomes, not all rRNA genes are actively transcribed. In budding yeast, only about 50% of the genes on average are transcribed in exponentially growing cells. Each ribosomal gene unit spreads over 9.1 kb of DNA and contains two transcribed regions encoding the 35S pre-rRNA, transcribed by RNA Polymerase I (Pol I), and the 5S rRNA, transcribed by Pol III (Figure 1A). These transcribed regions are separated by intergenic spacers (IGSs): IGS1 starts at the transcription termination site of the 35S gene and ends at the 5S rRNA gene terminator and IGS2 corresponds to the region between the 5S rRNA gene promoter and the promoter of the next 35S gene (Nomura, 2001). Pol I transcription accounts for almost 60% of total transcriptional activity in yeast cells (Warner, 1999). This process occurs in the nucleolus and results in the synthesis of the 35S pre-rRNA containing the sequences of three of the four rRNAs composing the mature ribosome, the 18S, 5.8S and 25S rRNAs. These sequences are flanked and separated by sequences that are not retained in the mature ribosomes: respectively the 5′ and 3′ external transcribed spacers (5′ ETS and 3′ ETS) and the internal transcribed spacers 1 and 2 (ITS1 and ITS2) (Figure 1A). This 35S precursor will be co-transcriptionally packaged into pre-ribosomal particles that will undergo a complex maturation pathway to generate the mature ribosomal subunits.

## Transcription Initiation and Termination

Pol I enzyme in yeast is composed of 14 subunits (global molecular weight of 590 kDa) including two large subunits, Rpa190 and Rpa135, jointly forming the active site of the enzyme (Riva et al., 1987) (Figure 1B). Crystal structure of yeast *Saccharomyces cerevisiae* Pol I revealed the interactions occurring between its 14 subunits: the two large subunits Rpa190 and Rpa135 organize the enzyme in two modules of similar mass (Engel et al., 2013; Fernández-Tornero et al., 2013). The Pol I-specific subunits whose role during transcription has been partially characterized include Rpa43 and Rpa14 subunits in the stalk, and Rpa34, Rpa49 and Rpa12 subunits associated with the jaw/lobe module (Figure 1B).

Formation of preinitiation complex (PIC) is presented in Figure 1C. Pol I promoter contains two sequences required for efficient transcription initiation: the upstream activating sequence (UAS) and the core element (CE) (Nomura, 2001; Boukhgalter et al., 2002). Recruitment of the polymerase to the promoter to form the PIC relies on four transcription factors: upstream activating factor (UAF), core factor (CF), TATA-binding protein (TBP) and the Rrn3 transcription factor (Keener et al., 1998). UAF is the first complex to associate with the UAS of the rDNA promoter to initiate PIC assembly (Steffan et al., 1996). TBP was shown to bind to both CF and UAF, thus serving as a bridge to position CF downstream of the UAS. Binding of CF to the CE allows further recruitment of Pol I stably associated with Rrn3 (Aprikian et al., 2001). Rrn3 is a highly conserved transcription factor that associates with the Rpa43-Rpa14 heterodimer of Pol I and interacts with the Rrn6 subunit of the CF. It is therefore a crucial element required for transcription initiation (Peyroche et al., 2000; Aprikian et al., 2001). Transcription begins at the transcription start site (TSS) and Pol I and Rrn3 are released from the PIC upon transcription initiation. Several structural studies gave new insights into Pol I promoter recognition and melting, and more broadly into transcription initiation by yeast Pol I (Blattner et al., 2011; Engel et al., 2013, 2016; Moreno-Morcillo et al., 2014; Neyer et al., 2016; Tafur et al., 2016; Han et al., 2017; Sadian et al., 2017; Smith et al., 2018; Sadian et al., 2019; Tafur et al., 2019; Knutson et al., 2020). These studies will not be detailed here.

Pol I transcription termination involves pausing induced by a terminator protein, leading to dissociation of the polymerase and release of the primary transcript. Paradoxically, termination is not required for rRNA production since nascent transcript is released through the endonucleolytic cleavage by Rnt1 (Figure 1D) (Henras et al., 2005). In fission yeast, Reb1 protein interacts with the Rpa12 subunit of Pol I to stimulate termination (Jaiswal et al., 2016). In budding yeast, 90% of Pol I transcription termination occurs at a well-defined primary terminator element (T1) downstream of the 25S rRNA sequence (Figure 1D). Transcription termination at this site implicates the DNA-binding factor Nsi1, a Reb1 paralog, which promotes termination upstream of T1 at a T-rich element that likely operates as a polymerase release element (Lang and Reeder, 1993; Merkl et al., 2014; Reiter et al., 2012). In 10% of the cases, Pol I reads through this first terminator and stops at a downstream, “fail-safe” terminator (T2) located around position +250 from the 3′ end of the 25S rRNA sequence (Reeder et al., 1999). Transcription termination on Pol II-transcribed genes was shown to involve the 5′-3′ exoribonuclease Rat1 through a mechanism called “torpedo” (West et al., 2004; Luo et al., 2006; Kim et al., 2004). According to this model, Rat1 binds and degrades the transcript emerging from the polymerase following cleavage and release of the pre-mRNA, and given its high processivity, Rat1 catches up and dissociates Pol II from the DNA template. In the context of Pol I transcription, Rat1 was shown to interact with terminator sequences T1 and T2 and to be required for efficient termination. Its catalytic activity is required for this function since expression of a catalytically inactive mutant of Rat1 (Rat1_D235A_) could not suppress the Pol I termination defect observed in absence of Rat1. The absence of both Rat1 and Fob1, bound to the replication fork barrier (RFB) site (Figure 1D), increases polymerase read-through of T2 and the RFB site, indicating that Fob1 is also partly involved in termination (El Hage et al., 2008).

## Pol I Subunits and Trans-acting Factors Involved in Elongation Dynamics

Transcription elongation properties involve in particular three Pol I subunits present on the lobe (Figure 1B): Rpa12 and the heterodimer Rpa34/Rpa49 (Liljelund et al., 1992; Nogi et al., 1993; Gadal et al., 1997). In absence of Rpa34/Rpa49, Pol I activity is altered (Huet et al., 1975; Liljelund et al., 1992). Pol I lacking the Rpa34/Rpa49 subunits does not produce RNA to the same extent as a wild-type enzyme (Kuhn et al., 2007; Beckouet et al., 2008; Albert et al., 2011). Furthermore, this heterodimer plays an important role in transcription by improving the recruitment of the Rrn3-Pol I complex to the rDNA and by triggering the release of Rrn3 from elongating Pol I. Indeed, in an *rpa49* deletion strain, Rrn3 is recruited less efficiently at the promoter and fails to dissociate from elongating polymerases following transcription initiation (Beckouet et al., 2008). Interestingly, Rpa49 and Rpa34 are important for nucleolar assembly and formation of a property of actively transcribed rRNA genes called “Pol I caravans” or “Pol I convoys,” reflecting a spatial proximity between adjacent polymerases (Albert et al., 2011; Neyer et al., 2016). Rpa12 subunit stabilizes the Rpa49/Rpa34 heterodimer on the polymerase (Van Mullem et al., 2002; Tafur et al., 2019). In the absence of Rpa12, Pol I catalytic properties are affected (Appling et al., 2018; Scull et al., 2021). Furthermore, Pol I transcription through a linear mono-nucleosomal template was shown to be defective in the absence of the lobe-binding subunits (Merkl et al., 2020). Mutations affecting the Rpa135 subunit were also shown to affect transcription elongation. In particular, mutation of the amino acid at position 784 (rpa135-D784G), suspected to play a role in loading NTP substrates, caused reduced transcription compared to a wild-type Pol I. Calculation of Pol I elongation rate *in vitro* showed that this Rpa135 mutant is ten times slower than the wild-type polymerase (Schneider et al., 2007).

In addition to the role of Pol I subunits in transcription elongation, transcription factor Spt5 in complex with Spt4, was also shown to be required for efficient Pol I transcription (Schneider et al., 2006). Immunoprecipitation and mass spectrometry experiments showed that this complex interacts directly with multiple Pol I subunits (Rpa49, Rpa34, Rpa135 and Rpa190), through the NGN and KOW domains of Spt5 (Schneider et al., 2006). Moreover, Spt5 also associates with the transcription factor Rrn3 and with the 35S rRNA gene (coding region and promoter) (Viktorovskaya et al., 2011). Depletion of Spt4 in yeast results in a temperature-sensitive slow growth phenotype associated with a decreased rRNA synthesis rate as well as a reduced Pol I elongation efficiency, also impacting pre-rRNA processing and ribosome assembly (Schneider et al., 2006). Furthermore, Spt5 mutations suppress the cold-sensitive phenotype of an *rpa49Δ* strain. All these data support a function of the Spt4-Spt5 complex in Pol I transcription elongation, which remains to be understood at the molecular level. Another related protein, Spt6, interacts with the Spt4/Spt5 complex and was also proposed to play a role in Pol I transcription (Swanson and Winston, 1992). Spt6 interacts with Pol I subunit Rpa43 (Beckouët et al., 2011). It was shown that Spt6 associates with rDNA and is required for Pol I transcription since a strain carrying an in-frame deletion allele of *SPT6 (Spt6-1004)* showed reduced Pol I occupancy on the rDNA (Engel et al., 2015). Other factors including Hmo1 also modulate Pol I elongation properties, but the underlying mechanisms remain elusive (Albert et al., 2013; Higashino et al., 2015).

## Mapping Pol I Position at Nucleotide Resolution to Investigate Pol I Elongation *In Vivo*

**FIGURE 2:**
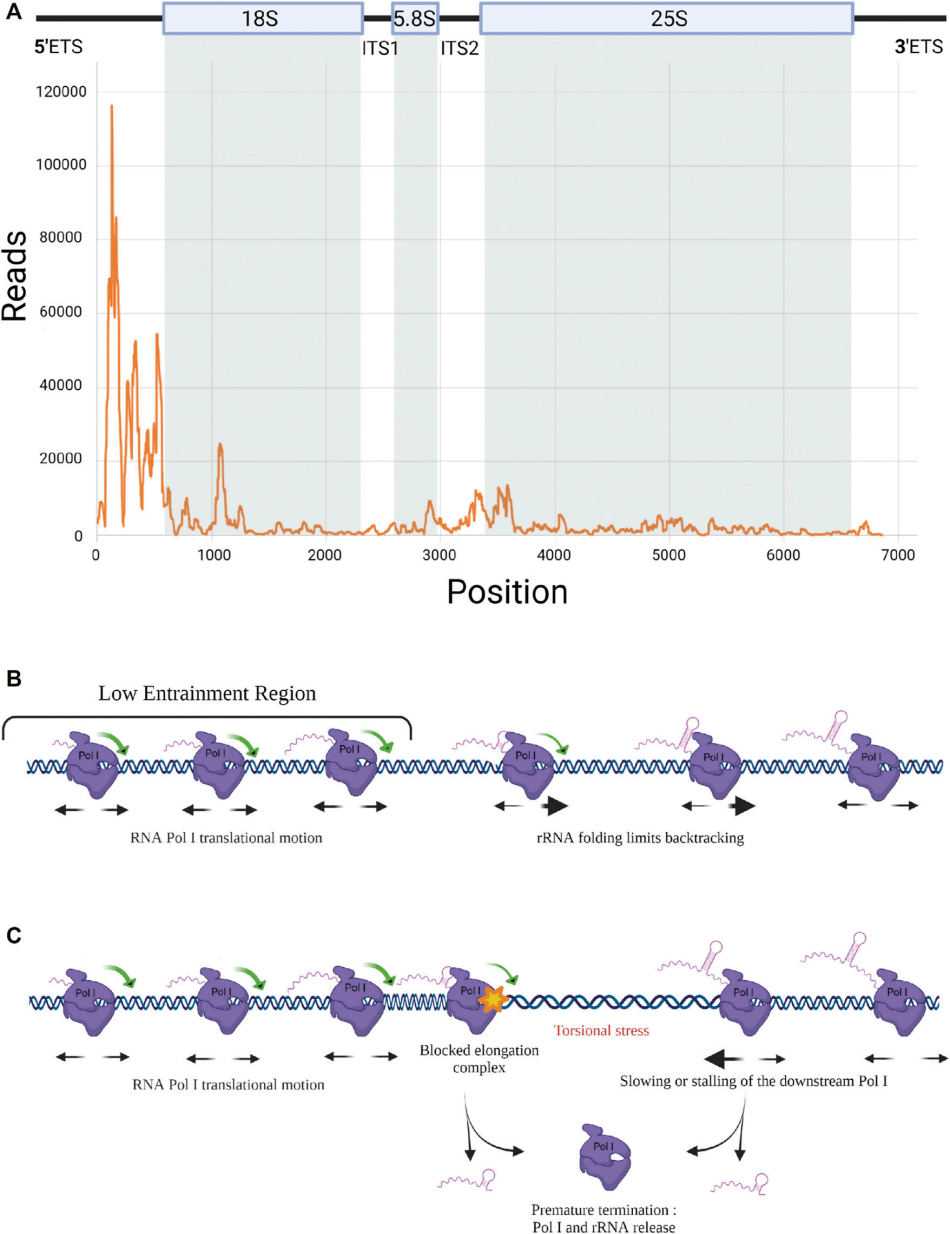
RNA Pol I elongation dynamics. **(A)** RNA Pol I distribution along rDNA template. Rpa135-CRAC results showing strong Pol I accumulation at 5’ end of the rRNA gene (Turowski et al., 2020). **(B)** Elongation dynamics in the Low Entrainment Region. Schematic representation of Pol I elongation dynamics in the LER (Turowski et al., 2020). Associated with short nascent transcripts, Pol I can easily rotate around rDNA in the 5’ region, leading to free translocation and a higher rate of backtracking. Beyond the LER, viscous drag limits the rotation of the pre-RNA/Pol I complex around DNA. **(C)** Premature termination. Model including the propensity of the elongation complex to dissociate and release rRNA, leading to premature termination. When a Pol I is stalled, a torsional stress occurs that could be resolved by a premature transcription termination (PTT) event.

In addition to the implication of Pol I subunits and trans-acting factors, Pol I elongation is also regulated by mechanisms intrinsic to the transcription process. Elongation is fundamentally discontinuous, with events of pausing, backtracking and possible premature termination, which remain to be explored. Pol I elongation was studied using the native elongating transcript sequencing (NET-seq) method, based on deep sequencing of the 3′ ends of nascent transcripts associated with the polymerase (Churchman and Weissman, 2011). This study revealed hundreds of positions within rDNA that reproducibly induce pausing (Clarke et al., 2018). Unfortunately, fragments of mature rRNAs co-purifying with Pol I in the NET-seq procedure could introduce bias in the analysis. Turowski and co-workers used the crosslinking and analysis of cDNAs (CRAC) technique to map the position of Pol I on rDNA during elongation. CRAC consists in crosslinking Pol I to its associated nascent rRNAs during elongation *in vivo*, followed by complex purification, reverse transcription of associated rRNAs and sequencing of cDNAs (Turowski et al., 2020). Applied to a population of cells, this method provides a statistical snapshot of the position of transcribing Pol I all along the rDNA unit and allows the determination of areas of the gene in which Pol I is accumulated (Figure 2A). It is noteworthy that a high polymerase occupancy reflects a low elongation rate. This CRAC analysis revealed a massive Pol I enrichment in the 5′ end of rRNA genes. Enrichment of polymerases at the 5′ end of rRNA genes was previously observed, but to a much lower extent, using the chromatin spread method developed by Oskar Miller, allowing a direct observation of Pol I in complex along rDNA (Miller and Beatty, 1969; Osheim et al., 2009; French et al., 2003). It was speculated that the high density of polymerases in the 5′ETS region, called “Low Entrainment Region” (LER), results in polymerases moving more slowly (decreasing Pol I elongation rate <20%) and being more closely over the initial 2 kb. As an underlying mechanism, Turowski and collaborators proposed that in the LER, where Pol I is associated with only short nascent transcripts, Pol I molecules are able to rotate freely along DNA grooves during elongation, while they become progressively unable to do so due to viscous drag 2 kb after initiation (Figure 2B). Accordingly, polymerase activity in the LER would not generate torsion in DNA, which allows changes in the relative positions of adjacent polymerases. This results in increased freedom for movement, likely increasing the probability of backtracking events, which would explain the accumulation of Pol I in the 5′ region of the genes. The high density of polymerases in the 5′ETS region is also correlated with the fact that major early pre-rRNA assembly events take place on the 5′ region of nascent rRNA (Chaker-Margot et al., 2017).

It is important to note that Pol I translocation is based on Brownian ratchet motion making elongation prone to frequent backtracking and potentially sensitive to quite modest forces (Dangkulwanich et al., 2013). Co-transcriptional folding of the nascent rRNA has direct consequences on elongation by preventing backtracking, thereby favoring productive elongation (Turowski et al., 2020). Any co-transcriptional association with the nascent transcript of trans-acting factors (UTPs, snoRNPs) should have the same stimulatory effect on transcription. With up to 200 transcribing Pol I per rRNA gene, each enzyme is influenced by its neighbors along the template directly through steric constraints. Indirectly, away of the LER predicted to occur only in the first 2 kb, torsional constraints on DNA plays a major role (torsional coupling):

- When the rotation around DNA of the transcribing polymerases is prevented by viscous drag due to the size and structure of nascent rRNA, elongation can be described within the twin-supercoiled domain model: DNA screws into the polymerase and experiences positive supercoiling downstream and negative supercoiling upstream (Liu and Wang, 1987).

- When all polymerases transcribe at the same rate, the negative DNA supercoiling created in the wake of one translocating polymerase is rapidly cancelled out by the positive DNA supercoiling created in front of the following one. The torsional stress between polymerases is alleviated and a fast and processive collective translocation is allowed, leading to polymerase convoys (Lesne et al., 2018; Kim et al., 2019). Therefore, all polymerases in convoys translocate at the same rate, their spacing remains constant (Figure 2C). Any change in the relative positions of transcribing polymerases generates torsional stress, which will quickly exceed the low stalling force of the polymerases (Ma et al., 2013; Heberling et al., 2016; Tantale et al., 2016). Any local modification of Pol I spacing within rDNA modifies DNA supercoiling, and the associated increase of local torsional energy generates an apparent force sufficiently strong to restore the initial distance between the polymerases and ensures the cohesion of the convoy (Lesne et al., 2018). Deletion or rapid depletion of topoisomerase I, results in defective rRNA synthesis (El Hage et al., 2010; Albert et al., 2019), highlighting the importance of resolving DNA supercoiling (downstream and upstream of each convoy) for efficient Pol I transcription elongation.

However, this cooperative long-distance group behavior may also induce antagonist effects on elongation. It was observed that promoter shut-off reduces the apparent elongation rate of the engaged polymerases, which is associated with a significant increase in premature termination (Kim et al., 2019). It is rational to suppose that the same effect occurs when elongating Pol I gets stalled on rDNA, thus leading to accumulation of negative torsional stress in the wake of the downstream Pol I (*i.e.* the nearby Pol I farthest from the promoter). Pol I stalling is known to increase premature termination of the paused Pol I and possibly also of the downstream polymerases (Figure 2C). Such a phenomenon was previously described as premature termination of transcription (PTT) for Pol II (Kamieniarz-Gdula and Proudfoot, 2019).

These premature termination events could also potentially explain the 5′ bias observed in the Pol I CRAC profile. Pol II is known to undergo a transition from initiation to elongation states that is associated with changes of the phosphorylation status of the C-terminal domain (CTD) of the largest Pol II subunit (Milligan et al., 2016). It is possible that Pol I undergoes a similar transition, the 5′ accumulation bias reflecting a region in which the polymerase has an elevated probability to terminate prematurely. However, consideration of premature termination in the model of Turowski and collaborators, even though it recapitulated the overall profile, reduced by 30% the total number of polymerases per transcription unit, which falls below the number of Pol I molecules per rDNA observed using Miller spreads (Turowski et al., 2020). Nevertheless, premature termination of Pol I cannot be excluded and could, at least partially, play a role in establishing the 5′ bias.

## Working Hypothesis: Pol I Processivity and Premature Termination

In order to better understand transcription regulation, Pol I mutants are of particular interest. We have recently identified in a genetic screen a super-active Pol I mutant, bearing a single substitution on the second largest subunit: Rpa135-F301S allele, hereafter named SuperPol I. This mutant induces an increase of rRNA production in yeast (Darrière et al., 2019). The mechanism leading to this increased rRNA production is not well understood. We proposed that this mutation alleviates an intrinsic repressive element of the polymerase, leading to increased processivity during elongation, *i.e*. the ability of Pol I to carry out continuous RNA synthesis on the DNA template without premature termination. This hypothesis is based on several experimental evidences. First, Miller spreads showed that the amounts of Pol I engaged in transcription are comparable in wild-type (WT) and mutant cells, meaning that the increased production of rRNA is not due to a major enhancement of Pol I initiation rate (Darrière et al., 2019). Moreover, *in vitro* promoter-dependent transcription assays confirmed that transcription initiation rate is similar between WT and SuperPol I. On the other hand, a tailed template assay, measuring elongation rate *in vitro*, revealed an increased rRNA production by the SuperPol I, likely due to a higher processivity (Darrière et al., 2019). Taken together, these elements suggest that the Rpa135-F301S mutation induces modifications in the elongation process, and more precisely on processivity. Premature termination directly affects processivity and likely influences Pol I distribution along the DNA template. Importantly, premature termination can not be measured by CRAC, which relies on detection of rRNA still bound to Pol I. To demonstrate the occurrence of premature termination events, defined as a dissociation of the elongation complex and release of the nascent rRNA, it will be necessary to correlate Pol I complex stalling with the production of abortive rRNAs. This could be achieved by combining Pol I CRAC data, highlighting precise pause sites, with a mapping of the corresponding abortive transcripts. Detection of rRNA species resulting from abortive transcription in differential amounts in cells expressing the SuperPol I or WT polymerase should allow to better understand what features of elongating Pol I lead to premature termination. The increased processivity of the SuperPol I mutant could likely be the consequence of a lower occurrence of premature termination, *i.e.* a lower production of abortive rRNAs.

## Conclusion and Perspectives

Methods allowing to map at nucleotide resolution Pol I pausing sites during elongation revealed a key interplay between RNA folding and elongation rate: formation of rRNA secondary structures prevents backtracking, hence enhances elongation rate. With a large amount of co-transcriptional folding of rRNA, we are now able to study how processing events are affecting Pol I elongation rate. So far limited to budding yeast, there is no doubt that some Pol I regulatory mechanisms are evolutionary conserved, as Pol I elongation rate is limiting for rRNA synthesis in metazoan cells (Hung et al., 2017). The understanding of the precise mechanisms of Pol I transcription and the implication of each inherent elongation feature opens wide prospects on health-related areas of research, particularly to understand a large number of genetic diseases collectively called ribosomopathies. Pol I inhibition used in cancer therapy these recent years will also benefit from such mechanistic breakthroughs (Sulima et al., 2019; Ferreira et al., 2020; Kampen et al., 2020).

## References

[B1] Albert B. Colleran C. Léger-Silvestre I. Berger A. B. Dez C. Normand C. (2013). Structure-function Analysis of Hmo1 Unveils an Ancestral Organization of HMG-Box Factors Involved in Ribosomal DNA Transcription from Yeast to Human. Nucleic Acids Res. 41, 10135–10149. 10.1093/nar/gkt770 24021628 PMC3905846

[B2] Albert B. Kos-Braun I. C. Henras A. Dez C. Rueda M. P. Zhang X. (2019). A Ribosome Assembly Stress Response Regulates Transcription to Maintain Proteome Homeostasis. bioRxiv [Preprint]. Available at: https://www.biorxiv.org/content/10.1101/512665v1.full . (Accessed January 6, 2019) 10.1101/512665 PMC6579557 31124783

[B3] Albert B. Léger-Silvestre I. Normand C. Ostermaier M. K. Pérez-Fernández J. Panov K. I. (2011). RNA Polymerase I-specific Subunits Promote Polymerase Clustering to Enhance the rRNA Gene Transcription Cycle. J. Cel Biol. 192, 277–293. 10.1083/jcb.201006040 PMC3172167 21263028

[B4] Appling F. D. Scull C. E. Lucius A. L. Schneider D. A. (2018). The A12.2 Subunit Is an Intrinsic Destabilizer of the RNA Polymerase I Elongation Complex. Biophysical J. 114, 2507–2515. 10.1016/j.bpj.2018.04.015 PMC6129170 29874602

[B5] Aprikian P. Moorefield B. Reeder R. H. (2001). New Model for the Yeast RNA Polymerase I Transcription Cycle. Mol. Cel. Biol. 21, 4847–4855. 10.1128/MCB.21.15.4847-4855.2001 PMC87188 11438642

[B6] Beckouet F. Labarre-Mariotte S. Albert B. Imazawa Y. Werner M. Gadal O. (2008). Two RNA Polymerase I Subunits Control the Binding and Release of Rrn3 during Transcription. Mol. Cel. Biol. 28, 1596–1605. 10.1128/MCB.01464-07 PMC2258765 18086878

[B7] Beckouët F. Mariotte-Labarre S. Peyroche G. Nogi Y. Thuriaux P. (2011). Rpa43 and its Partners in the Yeast RNA Polymerase I Transcription Complex. FEBS Lett. 585, 3355–3359. 10.1016/j.febslet.2011.09.011 21983101

[B8] Blattner C. Jennebach S. Herzog F. Mayer A. Cheung A. C. M. Witte G. (2011). Molecular Basis of Rrn3-Regulated RNA Polymerase I Initiation and Cell Growth. Genes Develop. 25, 2093–2105. 10.1101/gad.17363311 21940764 PMC3197207

[B9] Boukhgalter B. Liu M. Guo A. Tripp M. Tran K. Huynh C. (2002). Characterization of a Fission Yeast Subunit of an RNA Polymerase I Essential Transcription Initiation Factor, SpRrn7h/TAF I 68, that Bridges Yeast and Mammals: Association with SpRrn11h and the Core Ribosomal RNA Gene Promoter. Gene 291, 187–201. 10.1016/s0378-1119(02)00597-8 12095692

[B10] Chaker-Margot M. Barandun J. Hunziker M. Klinge S. (2017). Architecture of the Yeast Small Subunit Processome. Science 355, eaal1880. 10.1126/science.aal1880 27980088

[B11] Churchman L. S. Weissman J. S. (2011). Nascent Transcript Sequencing Visualizes Transcription at Nucleotide Resolution. Nature 469, 368–373. 10.1038/nature09652 21248844 PMC3880149

[B12] Clarke A. M. Engel K. L. Giles K. E. Petit C. M. Schneider D. A. (2018). NETSeq Reveals Heterogeneous Nucleotide Incorporation by RNA Polymerase I. Proc. Natl. Acad. Sci. USA 115, E11633–E11641. 10.1073/pnas.1809421115 30482860 PMC6294894

[B13] Dangkulwanich M. Ishibashi T. Liu S. Kireeva M. L. Lubkowska L. Kashlev M. (2013). Complete Dissection of Transcription Elongation Reveals Slow Translocation of RNA Polymerase II in a Linear Ratchet Mechanism. Elife 2, e00971. 10.7554/eLife.00971 24066225 PMC3778554

[B14] Darrière T. Pilsl M. Sarthou M.-K. Chauvier A. Genty T. Audibert S. (2019). Genetic Analyses Led to the Discovery of a Super-active Mutant of the RNA Polymerase I. Plos Genet. 15, e1008157. 10.1371/journal.pgen.1008157 31136569 PMC6555540

[B15] El Hage A. French S. L. Beyer A. L. Tollervey D. (2010). Loss of Topoisomerase I Leads to R-Loop-Mediated Transcriptional Blocks During Ribosomal RNA Synthesis. Genes Develop. 24, 1546–1558. 10.1101/gad.573310 20634320 PMC2904944

[B16] El Hage A. Koper M. Kufel J. Tollervey D. (2008). Efficient Termination of Transcription by RNA Polymerase I Requires the 5' Exonuclease Rat1 in Yeast. Genes Develop. 22, 1069–1081. 10.1101/gad.463708 18413717 PMC2335327

[B17] Engel C. Plitzko J. Cramer P. (2016). RNA Polymerase I-Rrn3 Complex at 4.8 Å Resolution. Nat. Commun. 7, 12129. 10.1038/ncomms12129 27418309 PMC4947163

[B18] Engel C. Sainsbury S. Cheung A. C. Kostrewa D. Cramer P. (2013). RNA Polymerase I Structure and Transcription Regulation. Nature 502, 650–655. 10.1038/nature12712 24153182

[B19] Engel K. L. French S. L. Viktorovskaya O. V. Beyer A. L. Schneider D. A. (2015). Spt6 Is Essential for rRNA Synthesis by RNA Polymerase I. Mol. Cel. Biol. 35, 2321–2331. 10.1128/MCB.01499-14 PMC4456441 25918242

[B20] Fernández-Tornero C. Moreno-Morcillo M. Rashid U. J. Taylor N. M. I. Ruiz F. M. Gruene T. (2013). Crystal Structure of the 14-subunit RNA Polymerase I. Nature 502, 644–649. 10.1038/nature12636 24153184

[B21] Ferreira R. Schneekloth J. S. Panov K. I. Hannan K. M. Hannan R. D. (2020). Targeting the RNA Polymerase I Transcription for Cancer Therapy Comes of Age. Cells 9, 266. 10.3390/cells9020266 PMC7072222 31973211

[B22] French S. L. Osheim Y. N. Cioci F. Nomura M. Beyer A. L. (2003). In Exponentially Growing *Saccharomyces cerevisiae* Cells, rRNA Synthesis Is Determined by the Summed RNA Polymerase I Loading Rate Rather Than by the Number of Active Genes. Mol. Cel. Biol. 23, 1558–1568. 10.1128/mcb.23.5.1558-1568.2003 PMC151703 12588976

[B23] Gadal O. Mariotte-Labarre S. Chedin S. Quemeneur E. Carles C. Sentenac A. (1997). A34.5, a Nonessential Component of Yeast RNA Polymerase I, Cooperates with Subunit A14 and DNA Topoisomerase I to Produce a Functional rRNA Synthesis Machine. Mol. Cel. Biol. 17, 1787–1795. 10.1128/mcb.17.4.1787 PMC232025 9121426

[B24] Han Y. Yan C. Nguyen T. H. D. Jackobel A. J. Ivanov I. Knutson B. A. (2017). Structural Mechanism of ATP-independent Transcription Initiation by RNA Polymerase I. Elife 6, e27414. 10.7554/eLife.27414 28623663 PMC5489313

[B25] Heberling T. Davis L. Gedeon J. Morgan C. Gedeon T. (2016). A Mechanistic Model for Cooperative Behavior of Co-transcribing RNA Polymerases. Plos Comput. Biol. 12, e1005069. 10.1371/journal.pcbi.1005069 27517607 PMC4982667

[B26] Henras A. K. Sam M. Hiley S. L. Wu H. Hughes T. R. Feigon J. (2005). Biochemical and Genomic Analysis of Substrate Recognition by the Double-Stranded RNA Binding Domain of Yeast RNase III. RNA 11, 1225–1237. 10.1261/rna.2760705 15987808 PMC1370806

[B27] Higashino A. Shiwa Y. Yoshikawa H. Kokubo T. Kasahara K. (2015). Both HMG Boxes in Hmo1 Are Essential for DNA Binding In Vitro and In Vivo. Biosci. Biotechnol. Biochem. 79, 384–393. 10.1080/09168451.2014.978258 25410521

[B28] Huet J. Buhler J. M. Sentenac A. Fromageot P. (1975). Dissociation of Two Polypeptide Chains from Yeast RNA Polymerase A. Proc. Natl. Acad. Sci. 72, 3034–3038. 10.1073/pnas.72.8.3034 1103135 PMC432913

[B29] Hung S. S. Lesmana A. Peck A. Lee R. Tchoubrieva E. Hannan K. M. (2017). Cell Cycle and Growth Stimuli Regulate Different Steps of RNA Polymerase I Transcription. Gene 612, 36–48. 10.1016/j.gene.2016.12.015 27989772

[B30] Jaiswal R. Choudhury M. Zaman S. Singh S. Santosh V. Bastia D. (2016). Functional Architecture of the Reb1-Ter Complex of *Schizosaccharomyces pombe* . Proc. Natl. Acad. Sci. USA 113, E2267–E2276. 10.1073/pnas.1525465113 27035982 PMC4843429

[B31] Kamieniarz-Gdula K. Proudfoot N. J. (2019). Transcriptional Control by Premature Termination: A Forgotten Mechanism. Trends Genet. 35, 553–564. 10.1016/j.tig.2019.05.005 31213387 PMC7471841

[B32] Kampen K. R. Sulima S. O. Vereecke S. De Keersmaecker K. (2020). Hallmarks of Ribosomopathies. Nucleic Acids Res. 48, 1013–1028. 10.1093/nar/gkz637 31350888 PMC7026650

[B33] Keener J. Josaitis C. A. Dodd J. A. Nomura M. (1998). Reconstitution of Yeast RNA Polymerase I Transcription In Vitro from Purified Components. J. Biol. Chem. 273, 33795–33802. 10.1074/jbc.273.50.33795 9837969

[B34] Kim M. Ahn S.-H. Krogan N. J. Greenblatt J. F. Buratowski S. (2004). Transitions in RNA Polymerase II Elongation Complexes at the 3′ Ends of Genes. EMBO J. 23, 354–364. 10.1038/sj.emboj.7600053 14739930 PMC1271760

[B35] Kim S. Beltran B. Irnov I. Jacobs-Wagner C. (2019). Long-Distance Cooperative and Antagonistic RNA Polymerase Dynamics via DNA Supercoiling. Cell 179, 106–119. 10.1016/j.cell.2019.08.033 31539491

[B36] Knutson B. A. Smith M. L. Belkevich A. E. Fakhouri A. M. (2020). Molecular Topology of RNA Polymerase I Upstream Activation Factor. Mol. Cel Biol 40. 10.1128/MCB.00056-20 PMC7296216 32253346

[B37] Kuhn C.-D. Geiger S. R. Baumli S. Gartmann M. Gerber J. Jennebach S. (2007). Functional Architecture of RNA Polymerase I. Cell 131, 1260–1272. 10.1016/j.cell.2007.10.051 18160037

[B38] Lang W. H. Reeder R. H. (1993). The REB1 Site Is an Essential Component of a Terminator for RNA Polymerase I in *Saccharomyces cerevisiae* . Mol. Cel. Biol. 13, 649–658. 10.1128/mcb.13.1.649 PMC358943 8417359

[B39] Lesne A. Victor J.-M. Bertrand E. Basyuk E. Barbi M. (2018). The Role of Supercoiling in the Motor Activity of RNA Polymerases. Methods Mol. Biol. 1805, 215–232. 10.1007/978-1-4939-8556-2_11 29971720

[B40] Liljelund P. Mariotte S. Buhler J. M. Sentenac A. (1992). Characterization and Mutagenesis of the Gene Encoding the A49 Subunit of RNA Polymerase A in *Saccharomyces cerevisiae* . Proc. Natl. Acad. Sci. 89, 9302–9305. 10.1073/pnas.89.19.9302 1409638 PMC50114

[B41] Liu L. F. Wang J. C. (1987). Supercoiling of the DNA Template During Transcription. Proc. Natl. Acad. Sci. 84, 7024–7027. 10.1073/pnas.84.20.7024 2823250 PMC299221

[B42] Luo W. Johnson A. W. Bentley D. L. (2006). The Role of Rat1 in Coupling mRNA 3'-end Processing to Transcription Termination: Implications for a Unified Allosteric-torpedo Model. Genes Develop. 20, 954–965. 10.1101/gad.1409106 16598041 PMC1472303

[B43] Ma J. Bai L. Wang M. D. (2013). Transcription Under Torsion. Science 340, 1580–1583. 10.1126/science.1235441 23812716 PMC5657242

[B44] Merkl P. E. Pilsl M. Fremter T. Schwank K. Engel C. Längst G. (2020). RNA Polymerase I (Pol I) Passage Through Nucleosomes Depends on Pol I Subunits Binding its Lobe Structure. J. Biol. Chem. 295, 4782–4795. 10.1074/jbc.RA119.011827 32060094 PMC7152749

[B45] Merkl P. Perez-Fernandez J. Pilsl M. Reiter A. Williams L. Gerber J. (2014). Binding of the Termination Factor Nsi1 to its Cognate DNA Site Is Sufficient to Terminate RNA Polymerase I Transcription In Vitro and to Induce Termination In Vivo. Mol. Cell Biol. 34, 3817–3827. 10.1128/MCB.00395-14 25092870 PMC4187712

[B46] Miller O. L. Beatty B. R. (1969). Visualization of Nucleolar Genes. Science 164, 955–957. 10.1126/science.164.3882.955 5813982

[B47] Milligan L. Huynh‐Thu V. A. Delan‐Forino C. Tuck A. Petfalski E. Lombraña R. (2016). Strand‐specific, High‐resolution Mapping of Modified RNA Polymerase II. Mol. Syst. Biol. 12, 874. 10.15252/msb.20166869 27288397 PMC4915518

[B48] Moreno-Morcillo M. Taylor N. M. I. Gruene T. Legrand P. Rashid U. J. Ruiz F. M. (2014). Solving the RNA Polymerase I Structural Puzzle. Acta Cryst. D Biol. Crystallogr. 70, 2570–2582. 10.1107/S1399004714015788 25286842 PMC4188003

[B49] Mullem V. V. Landrieux E. Vandenhaute J. Thuriaux P. (2002). Rpa12p, a Conserved RNA Polymerase I Subunit with Two Functional Domains. Mol. Microbiol. 43, 1105–1113. 10.1046/j.1365-2958.2002.02824.x 11918799

[B50] Neyer S. Kunz M. Geiss C. Hantsche M. Hodirnau V.-V. Seybert A. (2016). Structure of RNA Polymerase I Transcribing Ribosomal DNA Genes. Nature 540, 607–610. 10.1038/nature20561 27842382

[B51] Nogi Y. Yano R. Dodd J. Carles C. Nomura M. (1993). Gene RRN4 in *Saccharomyces cerevisiae* Encodes the A12.2 Subunit of RNA Polymerase I and Is Essential Only at High Temperatures. Mol. Cel. Biol. 13, 114–122. 10.1128/mcb.13.1.114-122.1993 PMC358891 8417319

[B52] Nomura M. (2001). Ribosomal RNA Genes, RNA Polymerases, Nucleolar Structures, and Synthesis of rRNA in the Yeast *Saccharomyces cerevisiae* . Cold Spring Harbor Symposia Quantitative Biol. 66, 555–566. 10.1101/sqb.2001.66.555 12762057

[B53] Osheim Y. N. French S. L. Sikes M. L. Beyer A. L. (2009). Electron Microscope Visualization of RNA Transcription and Processing in *Saccharomyces cerevisiae* by Miller Chromatin Spreading. Methods Mol. Biol. 464, 55–69. 10.1007/978-1-60327-461-6_4 18951179

[B54] Peyroche G. Milkereit P. Bischler N. Tschochner H. Schultz P. Sentenac A. (2000). The Recruitment of RNA Polymerase I on rDNA Is Mediated by the Interaction of the A43 Subunit with Rrn3. EMBO J. 19, 5473–5482. 10.1093/emboj/19.20.5473 11032814 PMC314014

[B55] Reeder R. H. Guevara P. Roan J. G. (1999). *Saccharomyces cerevisiae* RNA Polymerase I Terminates Transcription at the Reb1 Terminator In Vivo. Mol. Cel. Biol. 19, 7369–7376. 10.1128/mcb.19.11.7369 PMC84730 10523625

[B56] Reiter A. Hamperl S. Seitz H. Merkl P. Perez-Fernandez J. Williams L. (2012). The Reb1-Homologue Ydr026c/Nsi1 Is Required for Efficient RNA Polymerase I Termination in Yeast. EMBO J. 31, 3480–3493. 10.1038/emboj.2012.185 22805593 PMC3419925

[B57] Riva M. Schäffner A. R. Sentenac A. Hartmann G. R. Mustaev A. A. Zaychikov E. F. (1987). Active Site Labeling of the RNA Polymerases A, B, and C from Yeast. J. Biol. Chem. 262, 14377–14380. 10.1016/s0021-9258(18)47803-9 3667579

[B58] Sadian Y. Baudin F. Tafur L. Murciano B. Wetzel R. Weis F. (2019). Molecular Insight into RNA Polymerase I Promoter Recognition and Promoter Melting. Nat. Commun. 10, 5543. 10.1038/s41467-019-13510-w 31804486 PMC6895186

[B59] Sadian Y. Tafur L. Kosinski J. Jakobi A. J. Wetzel R. Buczak K. (2017). Structural Insights into Transcription Initiation by Yeast RNA Polymerase I. EMBO J. 36, 2698–2709. 10.15252/embj.201796958 28739580 PMC5599796

[B60] Schneider D. A. French S. L. Osheim Y. N. Bailey A. O. Vu L. Dodd J. (2006). RNA Polymerase II Elongation Factors Spt4p and Spt5p Play Roles in Transcription Elongation by RNA Polymerase I and rRNA Processing. Proc. Natl. Acad. Sci. 103, 12707–12712. 10.1073/pnas.0605686103 16908835 PMC1568913

[B61] Schneider D. A. Michel A. Sikes M. L. Vu L. Dodd J. A. Salgia S. (2007). Transcription Elongation by RNA Polymerase I Is Linked to Efficient rRNA Processing and Ribosome Assembly. Mol. Cel 26, 217–229. 10.1016/j.molcel.2007.04.007 PMC1927085 17466624

[B62] Scull C. E. Lucius A. L. Schneider D. A. (2021). The N-Terminal Domain of the A12.2 Subunit Stimulates RNA Polymerase I Transcription Elongation. Biophys. J. 120 (10), 1883–1893. 10.1016/j.bpj.2021.03.007 33737158 PMC8204343

[B63] Smith M. L. Cui W. Jackobel A. J. Walker-Kopp N. Knutson B. A. (2018). Reconstitution of RNA Polymerase I Upstream Activating Factor and the Roles of Histones H3 and H4 in Complex Assembly. J. Mol. Biol. 430, 641–654. 10.1016/j.jmb.2018.01.003 29357286 PMC9746128

[B64] Steffan J. S. Keys D. A. Dodd J. A. Nomura M. (1996). The Role of TBP in rDNA Transcription by RNA Polymerase I in *Saccharomyces cerevisiae*: TBP Is Required for Upstream Activation Factor-dependent Recruitment of Core Factor. Genes Develop. 10, 2551–2563. 10.1101/gad.10.20.2551 8895657

[B65] Sulima S. Kampen K. De Keersmaecker K. (2019). Cancer Biogenesis in Ribosomopathies. Cells 8, 229. 10.3390/cells8030229 PMC6468915 30862070

[B66] Swanson M. S. Winston F. (1992). SPT4, SPT5 and SPT6 Interactions: Effects on Transcription and Viability in *Saccharomyces cerevisiae* . Genetics 132, 325–336. 10.1093/genetics/132.2.325 1330823 PMC1205139

[B67] Tafur L. Sadian Y. Hanske J. Wetzel R. Weis F. Müller C. W. (2019). The Cryo-EM Structure of a 12-subunit Variant of RNA Polymerase I Reveals Dissociation of the A49-A34.5 Heterodimer and Rearrangement of Subunit A12.2. Elife 8, e43204. 10.7554/eLife.43204 30913026 PMC6435322

[B68] Tafur L. Sadian Y. Hoffmann N. A. Jakobi A. J. Wetzel R. Hagen W. J. H. (2016). Molecular Structures of Transcribing RNA Polymerase I. Mol. Cel 64, 1135–1143. 10.1016/j.molcel.2016.11.013 PMC5179497 27867008

[B69] Tantale K. Mueller F. Kozulic-Pirher A. Lesne A. Victor J.-M. Robert M.-C. (2016). A Single-Molecule View of Transcription Reveals Convoys of RNA Polymerases and Multi-Scale Bursting. Nat. Commun. 7, 12248. 10.1038/ncomms12248 27461529 PMC4974459

[B70] Turowski T. W. Petfalski E. Goddard B. D. French S. L. Helwak A. Tollervey D. (2020). Nascent Transcript Folding Plays a Major Role in Determining RNA Polymerase Elongation Rates. Mol. Cel 79, 488–503. 10.1016/j.molcel.2020.06.002 PMC7427326 32585128

[B71] Viktorovskaya O. V. Appling F. D. Schneider D. A. (2011). Yeast Transcription Elongation Factor Spt5 Associates with RNA Polymerase I and RNA Polymerase II Directly. J. Biol. Chem. 286, 18825–18833. 10.1074/jbc.M110.202119 21467036 PMC3099699

[B72] Warner J. R. (1999). The Economics of Ribosome Biosynthesis in Yeast. Trends Biochem. Sci. 24, 437–440. 10.1016/s0968-0004(99)01460-7 10542411

[B73] West S. Gromak N. Proudfoot N. J. (2004). Human 5′ → 3′ Exonuclease Xrn2 Promotes Transcription Termination at Co-transcriptional Cleavage Sites. Nature 432, 522–525. 10.1038/nature03035 15565158

